# Natural Immunity to HIV: A Delicate Balance between Strength and Control

**DOI:** 10.1155/2012/875821

**Published:** 2012-12-11

**Authors:** Johanne Poudrier, Valérie Thibodeau, Michel Roger

**Affiliations:** Laboratoire d'Immunogénétique, Centre de Recherche, Centre Hospitalier de l'Université de Montréal (CRCHUM) et Département de Microbiologie et Immunologie, Université de Montréal, Montreal, QC, Canada H2L 4M1

## Abstract

Understanding how the mucosal immune system in the human female reproductive tract might prevent or facilitate HIV infection has important implications for the design of effective interventions. We and others have established cohorts of highly-exposed, HIV-seronegative individuals, such as HIV-uninfected commercial sex workers, who have remained HIV-negative after more than 5 years of active prostitution. Observations obtained in studies of such individuals, who represent a model of natural immunity to HIV, indicate that HIV resistance may be associated with the host's capacity to preserve systemic integrity by constraining immune activity and controlling inflammatory conditions at the mucosal point of entry. This likely necessitates the orchestration of balanced, first-line and adaptive immune responses.

## 1. Introduction

At the end of 2010, 34 million people were living with HIV/AIDS world-wide. In that year, a total of 2.7 million people were infected by HIV, mostly through heterosexual intercourse, and 60% of new HIV infections affected women in sub-Saharan Africa [1]. Needless to say, the design of effective vaccines and microbicides to prevent HIV infection remains a global priority. High levels of anti-inflammatory and neutralizing proteins, such as antiproteases and HIV-specific immunoglobulins (Ig), are found in the genital mucosa of highly exposed HIV-seronegative (HESN) individuals, such as HIV-uninfected, “resistant” commercial sex workers (CSWs) [2, 3]. This suggests that efforts to develop effective microbicides and vaccines should aim at mimicking and/or soliciting innate and adaptive immune responses, such as those seen in the context of natural immunity to HIV. From such a viewpoint, vaccine approaches to specifically induced mucosal responses seem very promising. Indeed, genital IgA and IgG, elicited through combined intra-muscular and intranasal vaccination against HIV-gp41, delivered via virosome in nonhuman primates, prevented systemic HIV invasion by blocking transcytosis and by mediating antibody-dependent cellular cytotoxicity (ADCC) [4]. These animals lacked serum-neutralizing antibody activity, highlighting the role of effector antibodies at the mucosal point of entry, and their importance in preventing the dissemination of HIV infection [5]. In humans, the RV144 vaccine regimen (canarypox prime, HIV gp120 envelope (Env) glycoprotein boost) elicits protective responses, the nature of which remains to be defined in terms of generation and effector mechanisms [6]. Reduced rates of HIV acquisition without significant effects on initial viral loads or CD4 T-cell counts have led to the hypothesis of a transient, protective B-cell response. Moreover, binding of IgG antibodies to variable regions 1 and 2 (V1, V2) of Env has been shown to be inversely correlated with HIV infection rates [7]. Unfortunately, mucosal samples were not collected during the RV144 trial to assess mucosal Env-specific Ig levels, which we predict may constitute better correlates of protection. Success in conceiving effective vaccines most likely relies on their capacity to establish rapid, first-line immune responses at the mucosal point of entry as well as long-term protection, which operates both at the mucosal and systemic levels. 

A better understanding of the mechanisms of transmission and HIV-specific immune responses at the initial site of infection is therefore pivotal to the design of preventive strategies. Most observations relating to these events have been obtained with simian immunodeficiency virus (SIV) infection in nonhuman primates (reviewed in [8, 9]). In humans, findings in HESN individuals, such as HIV-uninfected CSWs, who represent a model of natural immunity to HIV, may thus yield important clues to the development of preventive approaches. As such, the current perspective on cumulative data, reported by us and others, supports the notion that HIV “resistance” in these highly exposed CSWs may be associated with their capacity to control genital inflammatory conditions and recruitment of HIV target cells at the initial site of infection. This could be achieved by locally constraining immune activity to mucosal sites and preserving peripheral integrity, a process that likely involves genetic factors and orchestration of strong innate and adaptive immune responses.

## 2. Immunology of the Female Genital (FGT)

**Figure 1:**
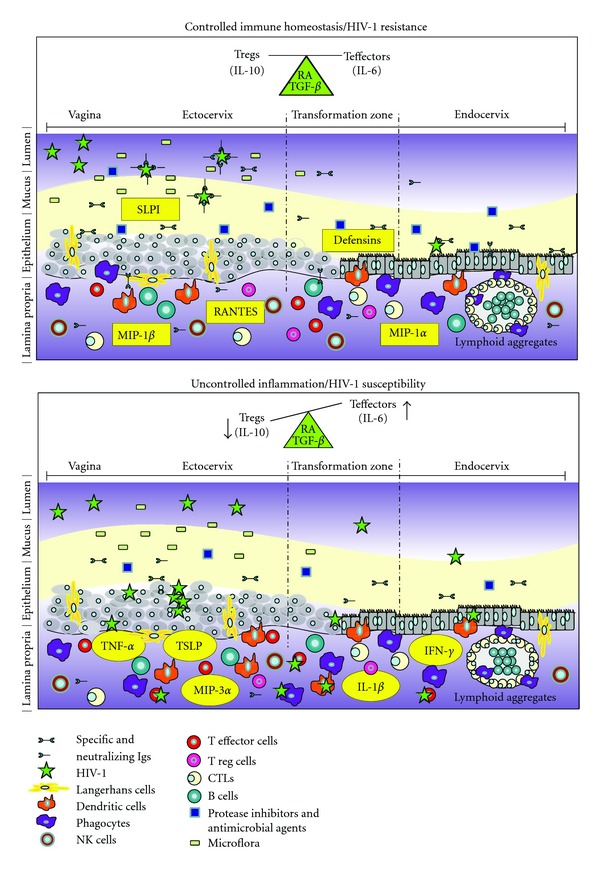
Qualitative and quantitative differences in mucosal innate and adaptive immune components are associated with the outcome of HIV infection in the female genital tract (FGT).

FGT immunology has been reviewed recently [10] and will only be summarized here briefly. The FGT is subdivided into 3 major areas presenting distinct phenotypic profiles: the nonsterile vagina and ectocervix colonized by commensal microflora, the sterile endometrium and fallopian tubes, and the endocervix in which sterility may be temporally related to menstrual cycle phase. Thus, FGT immunity is tightly regulated by a hormonal/inflammatory process throughout the menstrual cycle, having to deal with the pressure of procreation and microbial control. The innate immune compartment of the FGT involves the mucous lining of a tight epithelial cell (EC) barrier, stratified at the vaginal and ectocervical levels, as well as dendritic cells (DCs), Langerhans cells (LCs), macrophages, natural killer (NK) cells, and neutrophils, which confer protection through the production of antimicrobial agents, chemokines, and cytokines [10, 11] (Figure 1). Control of flora and invading pathogens is modulated via pattern recognition receptors (PRRs), such as toll-like receptors (TLRs) and NOD-like receptors, which recognise specific common microbial/pathogen-associated molecular patterns. As such, genital ECs form an uninterrupted barrier between the lumen and underlying cells and express PRRs, such as TLR-1 to -9, indicating the potential to respond to a wide range of microbes/pathogens [10–14]. DCs also express a large spectrum of PRRs, and the interaction between ECs and submucosal DCs likely modulates the maintenance of homeostatic balance between tolerance and inflammation in the FGT [10–15]. FGT-associated lymphoid organs are part of mucosal-associated lymphoid tissue, (MALT) which also includes gastrointestinal lymphoid tissue (GALT). Unlike GALT, the FGT does not contain M cells or organised lymphoid crypts or follicles in the submucosa. Rather, the upper FGT contains unique lymphoid aggregates constituted of CD8^+^ T cells that surround a central B cell core, which are encapsulated by macrophages [10] (Figure 1). Immunisation at the FGT level has been shown to elicit local CD8^+^ cytotoxic T lymphocyte (CTL), IgG, and IgA responses. Although immune induction mechanisms in the FGT remain poorly understood, it is likely that DCs migrate to FGT mucosal-associated lymphoid structures to induce first-line B-cell responses and to regulate adaptive lymphocyte responses [10, 16–18]. Interestingly, detailed characterisation of the Ig repertoire of cervical and systemic B cells from a HESN individual in Kenya disclosed that site-specific responses occur with unique regulation of tolerance and recruitment into local memory or blast B cell compartments. Also, the infusion of systemic post-germinal center B cells to the human cervix seems to be a common event [19]. These findings suggest that cervical B cell populations largely contribute to protection against HIV, by producing first-line and mature mucosal HIV-specific IgG and IgA, which are correlates of control “resistance” to HIV infection in the FGT of HESN women. Understanding how B cell populations are recruited and maintained in the FGT is crucial for the design of preventive approaches, to block infection by HIV at its main point of entry.

## 3. HIV Transmission in the FGT

Until now, the cascade of events leading to HIV infection after heterosexual transmission remains unclear. Several reports in humans and rhesus macaques suggest that LCs and DCs on mucosal surfaces are the earliest cell types to be exposed, and possibly infected by HIV or SIV, and migrate to the lamina propria and draining lymphoid tissues to facilitate transmission of the virus to permissive cells (reviewed in [8, 9, 20–23]). The most likely scenario has been proposed recently by the group of Haase [9, 24]. After genital administration to macaques, SIV establishes foci of infection in the vaginal sub-mucosa within a matter of days through a scheme involving macrophage inflammatory protein-3alpha (MIP-3*α*) (CCL20) production by ECs and early recruitment of interferon-alpha (IFN-*α*) producing plasmacytoid DC (pDC) as well as DC and CD4^+^ T-cell effectors, rapidly accessing draining lymph nodes and establishing systemic invasion by days 10–14. Recent studies with genital explants indicate that LCs can elaborate protrusions across the stratified epithelium into the lumen of the FGT to capture HIV mainly through the surface expression of langerin (CD207) [8, 20–23]. LCs may then enable HIV transmission to sub-mucosal DCs that express PRRs, such as DC-SIGN (CD209), and to CD4^+^ CCR5^+^ effector target T lymphocytes and/or migrate to draining lymphoid tissues. DCs are also thought to have the capacity to establish protrusions across the epithelium, enabling direct HIV transmission to permissive populations in the lamina propria or after their migration to draining lymphoid compartments [8, 20–23]. 

Although HIV does not productively infect ECs, it can be transcytosed, reaching sub-mucosal DC populations and effector target T lymphocytes in the lamina propria [25–30]. Furthermore, HIV likely facilitates its incursion through the genital epithelium by inducing a proinflammatory milieu that affects tight junction proteins and enhances microbial translocation [31]. HIV has also been shown to be internalized by FGT ECs via gp340, a scavenger receptor, subsequently promoting the production of proinflammatory thymic stromal lymphopoietin (TSLP) via TLR-7 signalling, which then activates DCs and promotes HIV transmission to CD4^+^ T cells [32, 33]. The galactosyl ceramide receptor induces HIV endocytosis in ECs and DCs, allowing transcytosis and transfer to susceptible CD4^+^ T cells [34].

HIV acquisition may depend on the level of inflammation and availability/permissiveness of target populations, such as activated CD4^+^ T cells expressing CCR5 and *α*4*β*7 [35, 36]. In human peripheral blood, CD4^+^ T cells expressing CCR6^+^ are most permissive to HIV infection [37, 38]. In the gut, which represents a major viral reservoir, mucosal Th17 effectors are the main targets of HIV/SIV [39–42]. These mucosal Th17 cells are mostly *α*4*β*7^+^ CD103^+^, express ROR*γ*t^+^ and have been reported to require factors, such as transforming growth factor-beta (TGF-*β*), interleukin (IL)-1, IL-6, IL-21, and IL-23, for their differentiation [43]. They express CCR6, a major ligand of MIP-3*α*, which is mainly secreted by mucosal ECs and is known to also attract immature LCs and DCs [44, 45]. It has been determined that homeostatic balance between mucosal T effector versus T regulatory (Treg) populations in the gut is modulated by EC and DC cross-talk, and is highly influenced by factors, such as retinoic acid (RA) and TGF-*β* [46, 47] (Figure 1). TGF-*β* is also known to influence FGT integrity [48, 49], and RA is involved in the regulation of ovarian function and FGT immune status by its modulatory effect on sexual hormones [50]. Also, oestrogen can upregulate RA and TGF-*β* production and signalling in the human endometrium [51, 52]. 

Thus, ECs and DCs appear to play a critical role in HIV infection by sensing through PRRs and orchestrating the dynamics of cellular populations, inflammatory conditions, and adaptive immune responses. The fact that TLR expression and responsiveness are increased in viraemic HIV infections suggests that TLR modulation is likely to influence HIV infection [53]. From this viewpoint, modulation of inflammatory responses through TLR agonists is a promising therapeutic approach in diseases with an imbalance in T cell responses, such as allergy and asthma, and could be seen as impacting inflammatory conditions and immune status in the FGT, the mucosal point of entry for the virus.

## 4. Nonpathogenic SIV Infections Provide Novel Insights into the Pathogenesis of Human HIV Infection 

Similar to pathogenic HIV and SIV infections in susceptible hosts, SIV infections in the natural host Sooty mangabeys result in high viral replication and massive depletion of gut mucosal effector CD4^+^ T cells [54]. However, a major distinction from pathogenic infection is the rapidly developing anti-inflammatory milieu that prevents chronic activation, apoptosis, and proliferation of T cells in SIV-infected Sooty mangabeys. This contributes to the maintenance of mucosal barrier integrity, preventing microbial translocation from the gut, which is the hallmark of pathogenic infections [55]. The control of disease progression appears to be linked to better management of aberrant immune activation caused by SIV infection. Indeed, the early onset of anti-inflammatory IL-10 production and Treg activity seems to be favoured in SIV nonpathogenic infections [54]. Furthermore, it was recently demonstrated that Sooty mangabeys generated less Th17 effector target cells than highly-susceptible macaques [40]. Importantly, the capacity to manage inflammatory conditions in Sooty mangabeys is associated with a low type I IFN gene profile. The latter appears to be linked to genetic polymorphisms in the type I IFN regulatory factor-7 (IRF-7) gene involved in the regulation of IFN production downstream of TLR-7 and -9 signalling, which are intracellular ligands for lentiviral ssRNA viruses, such as SIV and HIV, and CpG DNA, respectively [56]. Moreover, early blocking of MIP-3*α* and pro-inflammatory cytokines in the FGT of SIV-susceptible macaques prevented cellular recruitment, establishment of an inflammatory milieu, and infection despite repeated intravaginal exposure to high SIV doses [24]. Therefore, low inflammatory conditions are beneficial to the host in the context of HIV/SIV, and we believe that preventive approaches, such as microbicides, should be designed to induce and maintain a low inflammatory milieu.

## 5. Factors Associated with Susceptibility/Resistance to HIV Infection

The number of sexual partners and failure to use condoms are the best documented behavioural risk factors for sexual HIV transmission. Among the most compelling biological risk factors are the presence of vaginosis and sexually-transmitted infections, high viral load and low CD4^+^ T lymphocyte counts in infectious contact, and possibly viral virulence and tropism (reviewed in [57]). In Figure 1, top panel: controlled immune homeostasis results in resistance to HIV infection at the mucosal point of entry. Homeostatic balance between mucosal T effector versus T regulatory (Treg) populations is modulated by epithelial cell (EC) and dendritic cell (DC) cross-talk and is influenced by factors, such as retinoic acid (RA) and transforming growth factor-beta (TGF-*β*). Host factors associated with resistance to HIV infection involve the modulation of mucosal innate factors, such as defensins, secretory leukocyte proteaseinhibitor (SLPI), and other antiproteases as well as variations in frequencies and activities of DC, B, T, and natural killer (NK) cell populations [2, 58] (Table 1). HIV-specific mucosal IgA blocks viral transcytosis through the epithelium, and IgG is involved in antibody-dependent cellular cytotoxicity(ADCC). High levels of *β*-chemokines, such as macrophage inflammatory protein-1alpha (MIP-1*α*), MIP-1*β* and regulated upon activation, normal T-cell expressed and secreted (RANTES), which are natural CCR5 (major HIV coreceptor) ligands, can block cell viral entry to the FGT mucosa. Bottom panel: uncontrolled inflammation results in susceptibility to HIV infection at the mucosal point of entry. Unbalanced ratios between mucosal T effector versus Treg populations favouring high levels of T effectors are the hallmark of an inflammatory environment. Established vaginal inflammation can facilitate HIV infection through epithelium damage and recruitment of CD4^+^ T effectors, prime targets of HIV. The virus can also bind to ECs, be captured by Langerhans cells or DCs, and subsequently transcytosed and transferred to productively infect CD4^+^ target cells. Host factors associated with susceptibility to HIV infection are inflammatory markers, such as tumour necrosis factor-alpha (TNF-*α*), interferon-gamma (IFN-*γ*), IFN-*α*, interleukin-1beta (IL-1*β*), as well as thymic stromal lymphopoietin (TSLP) and MIP-3*α* secreted primarilyby ECs after downstream HIV signalling, favouring the recruitment of plasmacytoid DC (pDC) and CD4^+^ target cells. The promotion of an inflammatory milieu will contribute to infection and dissemination of HIV across the genital tract.

 NK cells represent a critical component of the host innate immune response against viral infections. The killing inhibitory receptor (KIR)3DL1/S1 locus has been linked with both slow progression to AIDS and resistance to HIV infection in a high-risk cohort of i.v. drug users from Montreal [59], and the KIR2DL2/DL3 locus has also been associated with resistance to HIV infection among African CSWs [60]. Functional modulation of NK cell responses (IFN-*γ*), NK activation (CD69), and degranulation (CD107a) markers has been correlated with resistance to HIV infection in several independent cohorts of HESN individuals [58]. Human leukocyte antigen (HLA) alleles, which are KIR ligands, are also associated with susceptibility/resistance to HIV infection and disease progression [61–65]. Other factors include IRF-1 [66], TLR-9 [67], and chemokine receptor/ligand polymorphisms, such as CCR5 [68–76], CCR2b [77, 78], CCL3 (MIP-1*α*) [79], and CCL4 (MIP-1*β*) [80]. Viral restriction factors, such as apolipoprotein B mRNA-editing catalytic polypeptide-like (APOBEC) 3G, tripartite motif (TRIM) 5*α*, tetherin, and sterile alpha motif and HD domain 1 (SAMHD1), exert anti-HIV activity. Lens epithelium-derived growth factor (LEDGF/p75) may also contribute to HIV resistance [81, 82]. Indeed, relatively low levels of LEDGF/p75 occurred in blood CD4^+^ T lymphocytes of HESN subjects enrolled in a Senegalese cohort of HIV-serodiscordant couples [83].

In a prospective cohort study of female CSWs in Nairobi, Kenya, over a 13-year period, a small group of women were found to be persistently IgG-seronegative and resistant to infection [102]. HIV resistance in this cohort has been associated with factors, such as trappin-2/elafin [84–86], serpins and cystatins in genital samples [87], certain HLA class I and II alleles [62], IRF-1 polymorphisms [66], and HIV-specific immune responses. Indeed, HIV-specific CD4^+^ T cell and CD8^+^ CTL responses as well as cross-clade neutralizing IgA have been encountered in both the blood and genital tract of resistant women [2, 88–94]. HIV-resistant CSWs from the Kenyan cohort had increased cervical CD4^+^ T cell counts compared to HIV-infected CSWs [92]. Moreover, CD4^+^ T cells in HIV-resistant women had a low activation profile but a much greater ability to proliferate in response to HIV p24 peptides than HIV-infected CSWs [93]. Also, in resistant women, higher levels of HIV-specific CTLs were noted in the cervix than in blood [89]. Recent studies have demonstrated that the quality of T cell responses in the context of HIV may be a major determinant of disease progression [95–97]. In a cohort of HESN women from the Ivory Coast, HIV-specific mucosal IgA was shown to block viral transcytosis through tight epithelial barriers [98, 99]. Thus, HIV-specific immune responses in CSWs prevail in the FGT and may be important in preventing heterosexual HIV infection. Interestingly, there is a clear indication of clustering of both resistance and HIV-specific CTL responses among HIV-resistant CSWs, suggesting that genetic factors could be involved in “protective” immune responses [103]. However, the durability and protective efficacy of CTL responses in these subjects are not absolute. Late seroconversion occurred in some HIV-resistant CSWs despite HIV-specific CTL responses [104]. Seroconversion happened in the absence of detectable CTL escape mutations and was related to the waning of HIV-specific CD8^+^ CTL responses due to reduced sexual activity and thus renewed antigenic exposure. These findings suggest that production and maintenance of HIV-specific effector responses and low-level immune activation may depend on genetically determined genital HIV-specific immune responses induced upon initial contact with HIV and low ongoing viral exposure. 

The recent finding that the frequency of immunosuppressive Treg lymphocytes was increased in the blood of HIV-resistant women [105] is consistent with the notion that the host's capacity to control and/or maintain low levels of immune activation may contribute to protection against infection. According to this view, in a cohort of highly HIV-exposed Beninese CSWs, we found that HIV-uninfected CSWs had significantly lower genital levels of tumour necrosis factor-alpha (TNF-*α*) and IFN-*γ* than HIV-infected CSWs [100] (Table 2). These observations suggest that the capacity to maintain a low-key inflammatory profile at the initial site of exposure is associated with protection against HIV infection in HESN individuals. In contrast, serum IL-2, IL-10, and TNF-*α* levels were significantly higher in HIV-uninfected CSWs than in HIV-infected CSWs. Importantly, when assessing the serum effector (IL-6) to regulatory (IL-10) ratio, we determined that it was lower in HIV-uninfected CSWs (0.9) than in HIV-infected CSWs (1.5). Moreover, the relatively normal cytokine levels found in the serum of HIV-uninfected CSWs (similar to nonexposed women from the general population) may be reflective of their capacity to maintain integrity of the systemic immune compartment by stopping HIV dissemination beyond the genital tract. In contrast, the low levels of cytokines observed in the serum of HIV-infected CSWs could reflect active recruitment of cytokine-producing cells to the genital mucosa in response to HIV infection. In this respect, HIV-infected CSWs had significantly higher blood and genital levels of monocyte chemotactic protein-3 (MCP-3) and monokine induced by gamma interferon (MIG) compared to both HIV-uninfected CSW and non-CSW groups [101]. In the HIV-infected group, MCP-3 and MIG levels were significantly higher in the genital mucosa than in blood, indicating a chemotactic gradient favouring the recruitment of immune cells contributing to the mucosal inflammatory response observed in these women. However, HIV-uninfected CSWs had significantly higher MIP-1*α* levels in the genital mucosa than both HIV-infected CSWs and HIV-uninfected non-CSW women. Moreover, serum MIP-1*α* (CCL3) and MIP-1*β* (CCL4) levels were higher in HIV-uninfected CSWs than the other groups. Interestingly, MIP-1*α* and MIP-1*β* are natural ligands of the HIV coreceptor CCR5, and high copy numbers of CCL3 and CCL4 genes have been previously associated with lower risk of HIV infection [79, 80], possibly by competing/blocking viral entry mediated by the co-receptor CCR5 [9]. Finally, recent evidence indicates that local microflora may also play a pivotal role in shaping host immune responses [2, 106], and thus may be a potential ally in the modulation of a mucosal immune compartment favourable to the maintenance of low inflammatory conditions. Thus, the immune events involved in natural immunity “resistance” to HIV may share some similarities with those associated with the control of the mucosal commensal microflora, which are thought to involve mucosal Igs and balanced Treg/T effector responses in absence of inflammation, and which cellular niches are maintained by repeated antigenic exposure, such as likely encountered by HESN individuals. 

## 6. Conclusion

Overall, resistance in the context of HIV infection may be associated with the host's capacity to induce a strong innate and HIV-specific immune response and, at the same time, control/maintain low inflammatory conditions and fewer HIV target cells at the initial exposure site (Figure 1). Understanding how the mucosal immune system in the human FGT might prevent or facilitate HIV infection has important implications for the design of effective interventions and may help develop strategies to modulate mucosal inflammatory conditions, to establish quick, long-lasting, first-line mucosal defence against HIV.

## Figures and Tables

**Table 1:** Genetic and genital mucosa host factors associated with resistance to HIV-1 infection in several HESN cohorts.

HIV-resistant host factors	
Genetic	Genital mucosa
KIR3D L1/S1 [59]	Protease inhibitors (SLPI, lactoferrin, serpins, cystatins, trappin-2/elafin) [2, 84–87]
KIR2D L2/L3 [60]	
HLA class 1 alleles [61–65]	Defensins (*α*, *β*) [2]
IRF-1 [66]	CC-*β* chemokines [9, 79, 80]
TLR 9 [67]	APOBEC3G, TRIM5*α*, tetherin, SAMHD1, LEDGF/p75 [81–83]
CCR5Δ32 [68–76]	Elevated DC and NK cell frequencies/activities [58]
CCR2b [77, 78]	CD4^+^- and CD8^+^-specific immune responses and reduced T-cell
MIP-1*α* [79]	activation [2, 88–97]
MIP-1*β* [80]	Cross-clade neutralizing specific IgA (transcytosis inhibition and ADCC activities) [2, 88–94, 98, 99]

ADCC: antibody-dependent cellular cytotoxicity; APOBEC: apolipoprotein B mRNA-editing catalytic polypeptide-like; CTL: cytotoxic T lymphocyte; DC: dendritic cell; HESN: highly-exposed HIV-seronegative; HLA: human leucocyte antigen; IRF: interferon-regulating factor; KIR: killing inhibitory receptor; LEDGF: Lens epithelium-derived growth factor; MIP: macrophage inflammatory protein; NK: natural killer; SAMHD: sterile alpha motif and HD domain; SLPI: secretory leukocyte protease inhibitor; TLR: Toll-like receptor; TRIM: tripartite motif.

**Table 2:** Cytokines and chemokines significantly associated with resistance to HIV-1 infection in the Beninese HIV-1-uninfected and infected CSW cohort.

Cytokines/chemokines	HIV-resistant CSWs	HIV-infected CSWs
Genital mucosa		
TNF-*α*	**↓**	**↑**
IFN-*γ*	**↓**	**↑**
MIP-1*α*	**↑↑**	**↓**
MCP-3	**↓**	**↑↑**
MIG	**↓**	**↑↑**
Blood		
IL-2	**↑**	**↓**
IL-10	**↑**	**↓**
TNF-*α*	**↑**	**↓**
MIP-1*α*	**↑**	**↓**
MIP-1*β*	**↑**	**↓**
MCP-3	**↓**	**↑**
MIG	**↓**	**↑**

[100, 101].

CSW: commercial sex worker; IL: interleukin; IFN: interferon; MCP: monocyte chemotactic protein; MIG: monokine induced by gamma interferon; MIP: macrophage inflammatory protein; TNF: tumour necrosis factor.
